# Crystal structures of (*Z*)-5-[2-(benzo[*b*]thio­phen-2-yl)-1-(3,5-di­meth­oxy­phen­yl)ethen­yl]-1*H*-tetra­zole and (*Z*)-5-[2-(benzo[*b*]thio­phen-3-yl)-1-(3,4,5-tri­meth­oxy­phen­yl)ethen­yl]-1*H*-tetra­zole

**DOI:** 10.1107/S2056989016005600

**Published:** 2016-04-08

**Authors:** Narsimha Reddy Penthala, Jaishankar K. B. Yadlapalli, Sean Parkin, Peter A. Crooks

**Affiliations:** a Department of Pharmaceutical Sciences, College of Pharmacy, University of Arkansas for Medical Sciences, Little Rock, AR 72205, USA; b Department of Chemistry, University of Kentucky, Lexington KY 40506, USA

**Keywords:** crystal structure, 5-substituted-1*H*-tetra­zoles, tetra­zole-tethered combretastatin A-4 analogs, anti­cancer agents, hydrogen bonding

## Abstract

(*Z*)-5-[2-(Benzo[*b*]thio­phen-2-yl)-1-(3,5-di­meth­oxy­phen­yl)ethen­yl]-1*H*-tetrazole methanol monosolvate, C_19_H_16_N_4_O_2_S·CH_3_OH, (I), was prepared by the reaction of (*Z*)-3-(benzo[*b*]thio­phen-2-yl)-2-(3,5-di­meth­oxy­phen­yl)acrylo­nitrile with tri­butyl­tin azide *via* a [3 + 2]cyclo­addition azide condensation reaction. The structurally related compound (*Z*)-5-[2-(benzo[*b*]thio­phen-3-yl)-1-(3,4,5-tri­meth­oxy­phen­yl)ethen­yl]-1*H*-tetra­zole, C_20_H_18_N_4_O_3_S, (II), was prepared by the reaction of (*Z*)-3-(benzo[*b*]thio­phen-3-yl)-2-(3,4,5-tri­meth­oxy­phen­yl)acrylo­nitrile with tri­butyl­tin azide. Crystals of (I) have two mol­ecules in the asymmetric unit (*Z*′ = 2), whereas crystals of (II) have *Z*′ = 1. The benzo­thio­phene rings in (I) and (II) are almost planar, with r.m.s deviations from the mean plane of 0.0084 and 0.0037 Å in (I) and 0.0084 Å in (II). The tetra­zole rings of (I) and (II) make dihedral angles with the mean planes of the benzo­thio­phene rings of 88.81 (13) and 88.92 (13)° in (I), and 60.94 (6)° in (II). The di­meth­oxy­phenyl and tri­meth­oxy­phenyl rings make dihedral angles with the benzo­thio­phene rings of 23.91 (8) and 24.99 (8)° in (I) and 84.47 (3)° in (II). In both structures, mol­ecules are linked into hydrogen-bonded chains. In (I), these chains involve both tetra­zole and methanol, and are parallel to the *b* axis. In (II), mol­ecules are linked into chains parallel to the *a* axis by N—H⋯N hydrogen bonds between adjacent tetra­zole rings.

## Abstract 2

In both structures, mol­ecules are linked into hydrogen-bonded chains. In (*Z*)-5-[2-(benzo[*b*]thio­phen-2-yl)-1-(3,5-di­meth­oxy­phen­yl)ethen­yl]-1*H*-tetra­zole methanol monosolvate, these chains involve both tetra­zole and methanol, and are parallel to the *b* axis. In (*Z*)-5-[2-(benzo[*b*]thio­phen-3-yl)-1-(3,4,5-tri­meth­oxy­phen­yl)ethen­yl]-1*H*-tetra­zole, mol­ecules are linked into chains parallel to the *a* axis by N—H⋯N hydrogen bonds between adjacent tetra­zole rings.

## Chemical context   

We have reported on benzo­thio­phene cyano­combretastatin A-4 analogs (Penthala *et al.*, 2013 ▸), and benzo­thio­phene triazol­ylcombretastatin A-4 analogs as promising anti-cancer agents (Penthala *et al.*, 2015 ▸). Previously, we published the synthesis of triazolylcombretastatin A-4 analogs utilizing a [3 + 2]cyclo­addition azide condensation reaction with sodium azide in the presence of l-proline as catalyst (Penthala *et al.*, 2014*a* ▸). In a continuation of our work on the chemical modification of the cyano group on the stilbene moiety of cyano­combretastatin A-4 analogs (Penthala *et al.*, 2014*a* ▸), we have recently synthesized tetra­zolylcombretastatin A-4 analogs as potential anti-cancer agents (Penthala *et al.*, 2016 ▸).

## Structural commentary   

**Figure 1:**
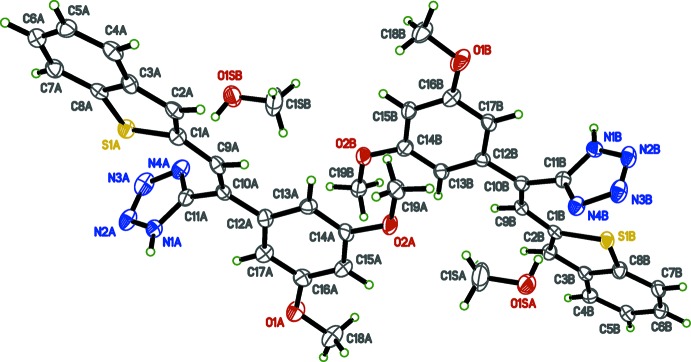
The mol­ecular structure of (I), with displacement ellipsoids drawn at the 50% probability level.

Single crystal X-ray analysis was carried out to obtain the structural conformations of the tetra­zolylcombretastatin A-4 analogs (I) and (II) for the analysis of structure–activity relationships (SAR), the relevance of the geometry of the tetra­zole ring on the stilbene scaffold and to confirm the position of the hydrogen atom in the tetra­zole ring system. The single crystal X-ray structures of (I) and (II) are shown in Figs. 1 ▸ and 2 ▸, respectively.
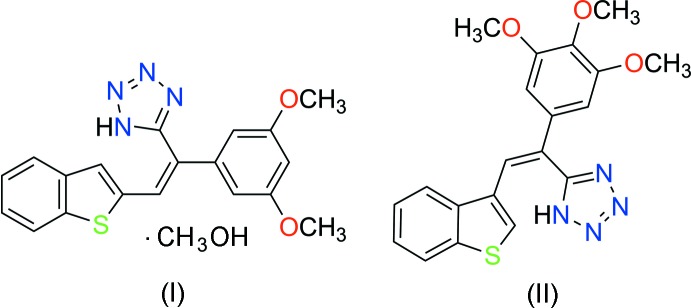


The benzo­thio­phene rings are almost planar with r.m.s. deviations from the mean plane of 0.0084 and 0.0037 Å in (I) and 0.0084 Å in (II), with bond distances and angles comparable with those reported for other benzo­thio­phene derivatives (Sonar *et al.*, 2007 ▸; Penthala *et al.*, 2014*b* ▸). The tetra­zole rings make dihedral angles with the mean plane of the benzo­thio­phene rings of 88.81 (13) and 88.92 (13)° in (I), and 60.94 (6)° in (II). The di­meth­oxy­phenyl ring in (I) and tri­meth­oxy­phenyl ring in (II) make dihedral angles with the benzo­thio­phene rings of 23.91 (8) and 24.99 (8)° in (I) and 84.47 (3)° in (II). Bond lengths and angles in both (I) and (II) are, by and large, unremarkable.

## Supra­molecular features   

**Figure 3:**
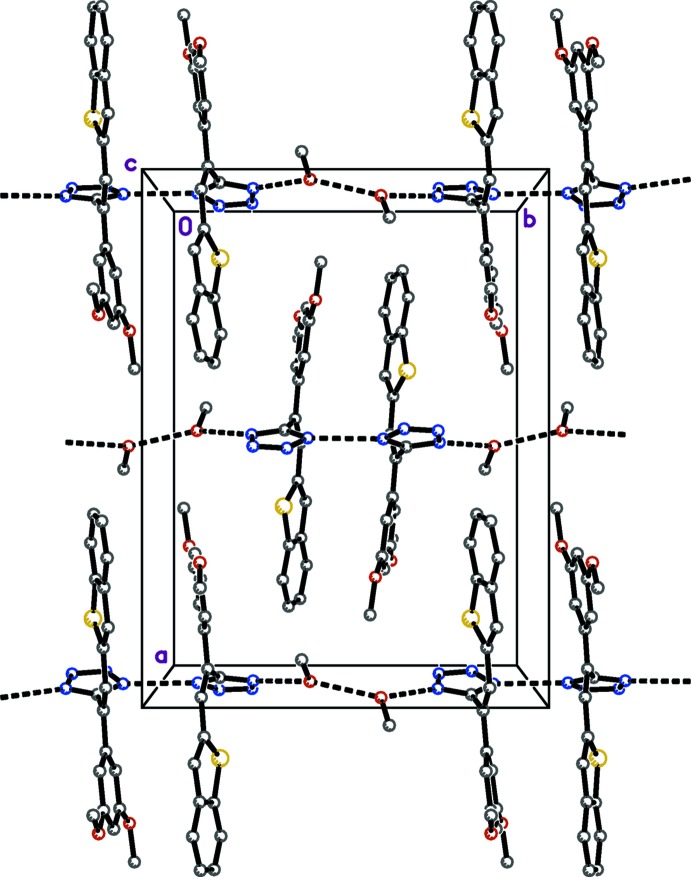
Crystal packing of (I), viewed down the *c* axis.

**Figure 4:**
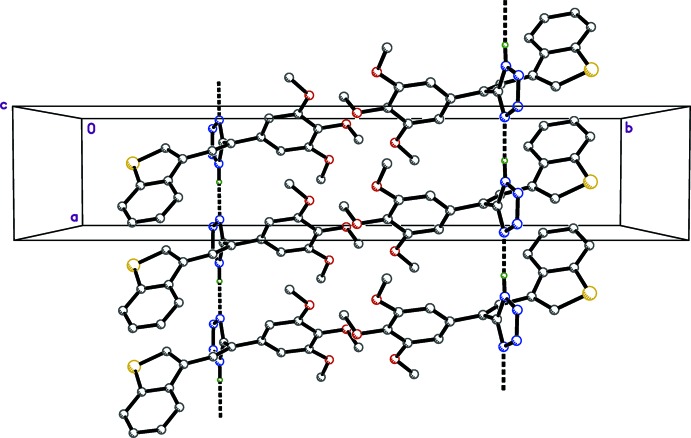
Crystal packing of (II), viewed down the *c* axis.

Hydrogen bonding and the mode of packing of (I) is illus­trated in Fig. 3 ▸, and the mode of packing of (II) is illustrated in Fig. 4 ▸. In the structure of (I), the mol­ecules are linked into hydrogen-bonded (Table 1 ▸) chains parallel to the crystallographic *b* axis involving inter­action between tetra­zole–tetra­zole (N—H⋯N), tetra­zole–methanol (O—H⋯N and N—H⋯O), and methanol–methanol (O—H⋯O). These chains are bidirectional, as the hydrogen atoms on the tetra­zole rings and the methanol oxygen atom appear to be disordered over two positions. In the structure of (II), the mol­ecules are linked into chains parallel to the *a* axis by inter­molecular N—H⋯N hydrogen bonds (Table 2 ▸) between adjacent tetra­zole rings.

## Database survey   

A search of the 2015 Cambridge Structural Database (Groom & Allen, 2014 ▸) for tetra­zole bonded *via* its carbon atom to another carbon atom yielded 255 hits. Of these, only two were bonded to an *sp* ^2^ carbon atom, namely 5-(2*H*-chromen-3-yl)-1*H*-tetra­zole monohydrate (NEYCUR: Gawande *et al.*, 2013 ▸) and (2*Z*,4*E*)-5-(di­methyl­amino)-2-(1*H*-tetra­zol-5-yl)penta-2,4-diene­nitrile methanol solvate (YUPPAB: Addicott *et al.*, 2009 ▸). Neither NEYCUR nor YUPPAB have any particular similarity to compounds (I) and (II).

## Synthesis and crystallization   

The title compounds (I) and (II) were prepared by utilizing our recently reported literature procedure (Penthala *et al.*, 2016 ▸). Recrystallization of the compounds from methanol afforded (I) and (II) as pale-yellow crystalline products which were suitable for X-ray analysis.

## Refinement details   

Crystal data, data collection and refinement details for both (I) and (II) are summarized in Table 3 ▸. H atoms were found in difference Fourier maps and subsequently placed at idealized positions with constrained distances of 0.95 Å (*R*_2_C*sp* ^2^H), 0.98 Å (*R*CH_3_), 0.84 Å (OH), 0.88 Å (N*sp* ^2^H). *U*_iso_(H) values were set to either 1.2*U*_eq_ or 1.5*U*_eq_ (*R*CH_3_, OH) of the attached atom. Final models were checked using *PLATON* (Spek, 2009 ▸), *RT* (Parkin, 2000 ▸), and by *checkCIF*.

Refinement of (I) was hampered by the presence of pseudosymmetry. An alternative model using space group *Pccn* was also refined, but the overall quality of the refinement was not as good as the *P*2_1_2_1_2 model given here. Indeed, the ADDSYM routine in *PLATON* (Spek, 2009 ▸) suggests a missing inversion centre and transformation to *Pccn*, but that model did not refine well (*R*_1_ > 9%). Other alternatives using space groups *Pcc*2, *Pban*, and *Pna*2_1_ were much less satisfactory. Not surprisingly, the *P*2_1_2_1_2 model was twinned by inversion, which was dealt with using standard *SHELXL* methods (TWIN and BASF commands).

The hydrogen on the tetra­zole ring was initially placed solely on the atoms labelled N1*A* and N1*B*. This assignment results in impossible clashes with symmetry equivalents about the twofold axis. Since there were suitable small difference map peaks for hydrogen atoms attached to atoms N4*A* and N4*B* as well as N1*A* and N1*B*, these hydrogen atoms were included as split over the two sites at half occupancy. Disorder of the tetra­zole ring hydrogen atoms in this way also requires that the hydroxyl hydrogen atoms of the methanol mol­ecules are disordered. Again, suitable (albeit small) difference map peaks were apparent. Further evidence for the disorder is that the distances C11*A*—N1*A*, C11*A*—N4*A* and C11*B*—N1*B*, C11*B*—N4*B* are all very similar, indicating that the C=N double bond and C—N single bond in these rings are scrambled. Not surprisingly, convergence of the OH hydrogen-atom positions was rather problematic.

## Supplementary Material

Crystal structure: contains datablock(s) global, I, II. DOI: 10.1107/S2056989016005600/hg5472sup1.cif

Structure factors: contains datablock(s) I. DOI: 10.1107/S2056989016005600/hg5472Isup2.hkl

Structure factors: contains datablock(s) II. DOI: 10.1107/S2056989016005600/hg5472IIsup3.hkl

CCDC references: 1472235, 1472234

Additional supporting information:  crystallographic information; 3D view; checkCIF report

## Figures and Tables

**Figure 2:**
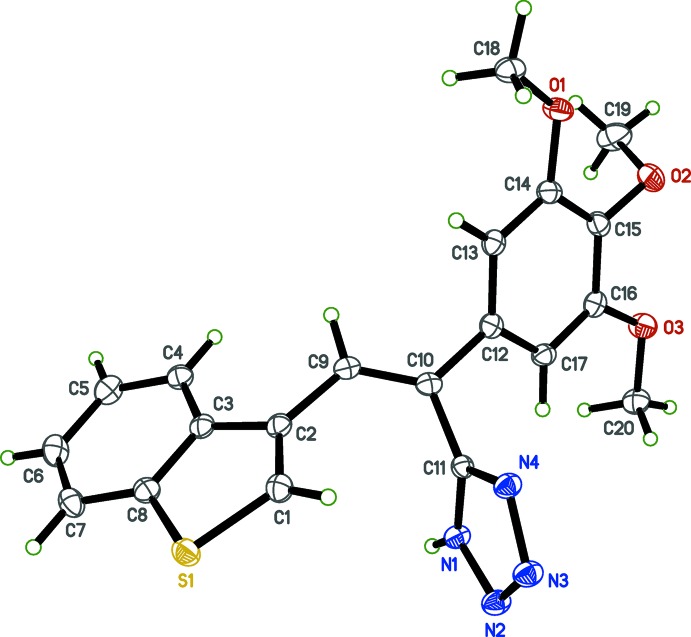
The mol­ecular structure of (II), with displacement ellipsoids drawn at the 50% probability level.

**Table 1:** Hydrogen-bond geometry (Å, °) for (I)

*D*—H⋯*A*	*D*—H	H⋯*A*	*D*⋯*A*	*D*—H⋯*A*
N1*A*—H1*NA*⋯N1*A* ^i^	0.88	1.91	2.787 (9)	176
N4*A*—H4*NA*⋯O1*SB*	0.88	1.87	2.736 (6)	168
N1*B*—H1*NB*⋯N1*B* ^ii^	0.88	1.92	2.792 (9)	174
N4*B*—H4*NB*⋯O1*SA*	0.88	1.91	2.769 (6)	165
O1*SA*—H1*SA*⋯N4*B*	0.84	1.97	2.769 (6)	158
O1*SA*—H2*SA*⋯O1*SA* ^iii^	0.84	1.81	2.646 (7)	177
O1*SB*—H1*SB*⋯N4*A*	0.84	1.90	2.736 (6)	176

Symmetry codes: (i) 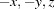; (ii) 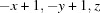; (iii) 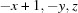.

**Table 2:** Hydrogen-bond geometry (Å, °) for (II)

*D*—H⋯*A*	*D*—H	H⋯*A*	*D*⋯*A*	*D*—H⋯*A*
N1—H1*N*⋯N3^i^	0.91 (2)	2.65 (2)	3.3886 (19)	138.5 (16)
N1—H1*N*⋯N4^i^	0.91 (2)	1.85 (2)	2.7482 (19)	167.1 (18)

Symmetry code: (i) 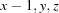.

**Table 3:** Experimental details

	(I)	(II)
Crystal data		
Chemical formula	C_19_H_16_N_4_O_2_S·CH_4_O	C_20_H_18_N_4_O_3_S
*M*_r_	396.46	394.44
Crystal system, space group	Orthorhombic, *P*2_1_2_1_2	Monoclinic, *P*2_1_/*c*
Temperature (K)	90	90
*a*, *b*, *c* (Å)	18.2226 (4), 13.7954 (5), 15.5594 (5)	4.8888 (1), 24.6650 (6), 15.5956 (4)
α, β, γ (°)	90, 90, 90	90, 91.031 (1), 90
*V* (Å^3^)	3911.4 (2)	1880.25 (8)
*Z*	8	4
Radiation type	Cu *K*α	Cu *K*α
μ (mm^−1^)	1.72	1.78
Crystal size (mm)	0.21 × 0.15 × 0.12	0.10 × 0.08 × 0.02

Data collection		
Diffractometer	Bruker X8 Proteum	Bruker X8 Proteum
Absorption correction	Multi-scan (*SADABS*; Krause *et al.*, 2015 ▸)	Multi-scan (*SADABS*; Krause *et al.*, 2015 ▸)
*T*_min_, *T*_max_	0.720, 0.915	0.693, 0.897
No. of measured, independent and observed [*I* > 2σ(*I*)] reflections	51755, 7112, 6916	23250, 3337, 3138
*R*_int_	0.038	0.037
(sin θ/λ)_max_ (Å^−1^)	0.602	0.603

Refinement		
*R*[*F* ^2^ > 2σ(*F* ^2^)], *wR*(*F* ^2^), *S*	0.042, 0.109, 1.10	0.034, 0.094, 1.13
No. of reflections	7112	3337
No. of parameters	514	259
H-atom treatment	H-atom parameters constrained	H atoms treated by a mixture of independent and constrained refinement
Δρ_max_, Δρ_min_ (e Å^−3^)	0.33, −0.34	0.27, −0.31
Absolute structure	Refined as an inversion twin	–
Absolute structure parameter	0.50 (3)	–

Computer programs: *APEX2* and *SAINT* (Bruker, 2006 ▸), *SHELXT* (Sheldrick, 2015*a* ▸), *SHELXL2014* (Sheldrick, 2015*b* ▸), *XP* in *SHELXTL* (Sheldrick, 2008 ▸) and *CIFFIX* (Parkin, 2013 ▸).

## supplementary crystallographic information

### (I) (Z)-5-[2-(Benzo[b]thiophen-2-yl)-1-(3,5-dimethoxyphenyl)ethenyl]-1H-tetrazole methanol monosolvate Crystal data

**Table:**

C_19_H_16_N_4_O_2_S·CH_4_O	*D*_x_ = 1.346 Mg m^−^^3^
*M**_r_* = 396.46	Cu *K*α radiation, λ = 1.54178 Å
Orthorhombic, *P*2_1_2_1_2	Cell parameters from 9871 reflections
*a* = 18.2226 (4) Å	θ = 4.9–68.2°
*b* = 13.7954 (5) Å	µ = 1.72 mm^−^^1^
*c* = 15.5594 (5) Å	*T* = 90 K
*V* = 3911.4 (2) Å^3^	Irregular block, pale yellow
*Z* = 8	0.21 × 0.15 × 0.12 mm
*F*(000) = 1664	

### (I) (Z)-5-[2-(Benzo[b]thiophen-2-yl)-1-(3,5-dimethoxyphenyl)ethenyl]-1H-tetrazole methanol monosolvate Data collection

**Table:**

Bruker X8 Proteum diffractometer	7112 independent reflections
Radiation source: fine-focus rotating anode	6916 reflections with *I* > 2σ(*I*)
Detector resolution: 5.6 pixels mm^-1^	*R*_int_ = 0.038
φ and ω scans	θ_max_ = 68.2°, θ_min_ = 2.8°
Absorption correction: multi-scan (*SADABS*; Krause *et al.*, 2015)	*h* = −21→17
*T*_min_ = 0.720, *T*_max_ = 0.915	*k* = −16→15
51755 measured reflections	*l* = −18→16

### (I) (Z)-5-[2-(Benzo[b]thiophen-2-yl)-1-(3,5-dimethoxyphenyl)ethenyl]-1H-tetrazole methanol monosolvate Refinement

**Table:**

Refinement on *F*^2^	Secondary atom site location: difference Fourier map
Least-squares matrix: full	Hydrogen site location: difference Fourier map
*R*[*F*^2^ > 2σ(*F*^2^)] = 0.042	H-atom parameters constrained
*wR*(*F*^2^) = 0.109	*w* = 1/[σ^2^(*F*_o_^2^) + (0.0327*P*)^2^ + 4.5187*P*] where *P* = (*F*_o_^2^ + 2*F*_c_^2^)/3
*S* = 1.10	(Δ/σ)_max_ < 0.001
7112 reflections	Δρ_max_ = 0.33 e Å^−^^3^
514 parameters	Δρ_min_ = −0.34 e Å^−^^3^
0 restraints	Absolute structure: Refined as an inversion twin
Primary atom site location: structure-invariant direct methods	Absolute structure parameter: 0.50 (3)

### (I) (Z)-5-[2-(Benzo[b]thiophen-2-yl)-1-(3,5-dimethoxyphenyl)ethenyl]-1H-tetrazole methanol monosolvate Special details

**Table:**

Experimental. The crystal was mounted using polyisobutene oil on the tip of a fine glass fibre, which was fastened in a copper mounting pin with electrical solder. It was placed directly into the cold gas stream of a liquid-nitrogen based cryostat. Diffraction data were collected with the crystal at 90K, which is standard practice in this laboratory for the majority of flash-cooled crystals.
Geometry. All esds (except the esd in the dihedral angle between two l.s. planes) are estimated using the full covariance matrix. The cell esds are taken into account individually in the estimation of esds in distances, angles and torsion angles; correlations between esds in cell parameters are only used when they are defined by crystal symmetry. An approximate (isotropic) treatment of cell esds is used for estimating esds involving l.s. planes.
Refinement. Refined as a 2-component inversion twin.

### (I) (Z)-5-[2-(Benzo[b]thiophen-2-yl)-1-(3,5-dimethoxyphenyl)ethenyl]-1H-tetrazole methanol monosolvate Fractional atomic coordinates and isotropic or equivalent isotropic displacement parameters (Å^2^)

**Table:**

	*x*	*y*	*z*	*U*_iso_*/*U*_eq_	Occ. (<1)
S1A	−0.14677 (5)	0.17392 (8)	0.08523 (7)	0.0251 (2)	
O1A	0.27407 (16)	0.0780 (3)	−0.00263 (19)	0.0339 (8)	
O2A	0.25387 (16)	0.1201 (3)	0.2996 (2)	0.0318 (8)	
N1A	−0.00422 (19)	0.1008 (3)	−0.0368 (2)	0.0246 (8)	
H1NA	−0.0012	0.0373	−0.0338	0.030*	0.5
N2A	−0.0252 (2)	0.1531 (3)	−0.1071 (2)	0.031 (1)	
N3A	−0.0231 (2)	0.2448 (3)	−0.0848 (3)	0.0349 (10)	
N4A	−0.00020 (19)	0.2525 (3)	−0.0020 (3)	0.0287 (8)	
H4NA	0.0062	0.3063	0.0274	0.034*	0.5
C1A	−0.0953 (2)	0.1363 (3)	0.1756 (3)	0.0223 (9)	
C2A	−0.1397 (2)	0.1174 (3)	0.2428 (3)	0.0231 (9)	
H2A	−0.1214	0.0973	0.2971	0.028*	
C3A	−0.2158 (2)	0.1295 (3)	0.2268 (3)	0.0239 (9)	
C4A	−0.2763 (2)	0.1176 (3)	0.2813 (3)	0.0244 (9)	
H4A	−0.2688	0.0980	0.3391	0.029*	
C5A	−0.3458 (2)	0.1339 (3)	0.2521 (3)	0.0277 (10)	
H5A	−0.3865	0.1249	0.2894	0.033*	
C6A	−0.3574 (2)	0.1642 (3)	0.1660 (3)	0.0273 (10)	
H6A	−0.4060	0.1742	0.1457	0.033*	
C7A	−0.2992 (2)	0.1793 (3)	0.1117 (3)	0.0270 (9)	
H7A	−0.3068	0.2022	0.0548	0.032*	
C8A	−0.2287 (2)	0.1604 (3)	0.1415 (3)	0.0226 (9)	
C9A	−0.0161 (2)	0.1270 (3)	0.1772 (3)	0.0204 (8)	
H9A	0.0036	0.1098	0.2316	0.025*	
C10A	0.0341 (2)	0.1385 (3)	0.1153 (3)	0.0211 (9)	
C11A	0.0106 (2)	0.1620 (4)	0.0259 (3)	0.0225 (9)	
C12A	0.1135 (2)	0.1267 (3)	0.1272 (3)	0.0224 (9)	
C13A	0.1443 (2)	0.1325 (3)	0.2116 (3)	0.0234 (9)	
H13A	0.1143	0.1463	0.2600	0.028*	
C14A	0.2187 (2)	0.1177 (3)	0.2212 (3)	0.0233 (9)	
C15A	0.2651 (2)	0.1003 (3)	0.1514 (3)	0.0271 (10)	
H15A	0.3163	0.0915	0.1594	0.033*	
C16A	0.2347 (2)	0.0964 (3)	0.0717 (3)	0.0268 (10)	
C17A	0.1596 (2)	0.1100 (3)	0.0598 (3)	0.0264 (10)	
H17A	0.1401	0.1077	0.0032	0.032*	
C18A	0.3507 (2)	0.0617 (4)	0.0066 (3)	0.0377 (12)	
H18A	0.3733	0.1180	0.0344	0.057*	
H18B	0.3728	0.0520	−0.0501	0.057*	
H18C	0.3588	0.0039	0.0420	0.057*	
C19A	0.2093 (3)	0.1288 (4)	0.3753 (3)	0.0335 (11)	
H19A	0.1845	0.1919	0.3752	0.050*	
H19B	0.2404	0.1236	0.4265	0.050*	
H19C	0.1725	0.0769	0.3759	0.050*	
S1B	0.63976 (5)	0.33164 (8)	0.41542 (7)	0.0245 (2)	
O1B	0.21860 (16)	0.4196 (3)	0.5019 (2)	0.0393 (9)	
O2B	0.23920 (16)	0.3710 (3)	0.2009 (2)	0.0306 (7)	
N1B	0.49906 (19)	0.3988 (3)	0.5359 (2)	0.0250 (8)	
H1NB	0.4969	0.4625	0.5332	0.030*	0.5
N2B	0.5193 (2)	0.3455 (3)	0.6043 (2)	0.0321 (10)	
N3B	0.5154 (2)	0.2549 (3)	0.5866 (3)	0.0332 (9)	
N4B	0.49224 (19)	0.2483 (3)	0.5038 (3)	0.0260 (8)	
H4NB	0.4848	0.1940	0.4754	0.031*	0.5
C1B	0.5881 (2)	0.3683 (3)	0.3263 (3)	0.0218 (9)	
C2B	0.6327 (2)	0.3889 (3)	0.2573 (3)	0.0244 (9)	
H2B	0.6144	0.4095	0.2031	0.029*	
C3B	0.7093 (2)	0.3762 (3)	0.2746 (3)	0.0215 (9)	
C4B	0.7694 (2)	0.3905 (3)	0.2191 (3)	0.0252 (9)	
H4B	0.7625	0.4114	0.1615	0.030*	
C5B	0.8392 (2)	0.3730 (3)	0.2517 (3)	0.0247 (9)	
H5B	0.8806	0.3819	0.2153	0.030*	
C6B	0.8503 (2)	0.3429 (3)	0.3357 (3)	0.0271 (10)	
H6B	0.8989	0.3321	0.3556	0.032*	
C7B	0.7919 (2)	0.3283 (3)	0.3910 (3)	0.0261 (9)	
H7B	0.7996	0.3076	0.4485	0.031*	
C8B	0.7214 (2)	0.3451 (3)	0.3595 (3)	0.0253 (9)	
C9B	0.5091 (2)	0.3757 (4)	0.3228 (3)	0.0233 (9)	
H9B	0.4895	0.3923	0.2681	0.028*	
C10B	0.4590 (2)	0.3625 (3)	0.3856 (3)	0.0211 (9)	
C11B	0.4828 (2)	0.3364 (3)	0.4728 (3)	0.0221 (9)	
C12B	0.3782 (2)	0.3708 (3)	0.3707 (3)	0.0200 (8)	
C13B	0.3489 (2)	0.3638 (3)	0.2897 (3)	0.0224 (9)	
H13B	0.3799	0.3513	0.2419	0.027*	
C14B	0.2740 (2)	0.3750 (3)	0.2778 (3)	0.0261 (10)	
C15B	0.2278 (2)	0.3939 (3)	0.3476 (3)	0.0249 (9)	
H15B	0.1765	0.4019	0.3390	0.030*	
C16B	0.2576 (2)	0.4008 (3)	0.4304 (3)	0.0261 (10)	
C17B	0.3329 (2)	0.3884 (3)	0.4430 (3)	0.0233 (9)	
H17B	0.3534	0.3918	0.4991	0.028*	
C18B	0.1415 (2)	0.4338 (4)	0.4934 (3)	0.0382 (12)	
H18D	0.1322	0.4897	0.4560	0.057*	
H18E	0.1200	0.4457	0.5501	0.057*	
H18F	0.1192	0.3758	0.4681	0.057*	
C19B	0.2836 (3)	0.3658 (4)	0.1264 (3)	0.0357 (12)	
H19D	0.3218	0.4157	0.1292	0.054*	
H19E	0.2531	0.3764	0.0753	0.054*	
H19F	0.3065	0.3017	0.1230	0.054*	
C1SA	0.4356 (4)	0.1141 (5)	0.3215 (5)	0.069 (2)	
H1S1	0.3850	0.1165	0.3428	0.103*	
H1S2	0.4399	0.0632	0.2779	0.103*	
H1S3	0.4486	0.1768	0.2961	0.103*	
O1SA	0.4839 (2)	0.0935 (2)	0.3908 (3)	0.0400 (9)	
H1SA	0.4764	0.1330	0.4310	0.060*	0.5
H2SA	0.4955	0.0346	0.3897	0.060*	0.5
C1SB	0.0583 (3)	0.3839 (4)	0.1772 (5)	0.065 (2)	
H1S4	0.0789	0.4438	0.2009	0.098*	
H1S5	0.0982	0.3422	0.1567	0.098*	
H1S6	0.0305	0.3499	0.2219	0.098*	
O1SB	0.0113 (2)	0.4064 (3)	0.1081 (2)	0.0391 (9)	
H1SB	0.0067	0.3576	0.0763	0.059*	0.5
H2SB	0.0327	0.4446	0.0743	0.059*	0.5

### (I) (Z)-5-[2-(Benzo[b]thiophen-2-yl)-1-(3,5-dimethoxyphenyl)ethenyl]-1H-tetrazole methanol monosolvate Atomic displacement parameters (Å^2^)

**Table:**

	*U*^11^	*U*^22^	*U*^33^	*U*^12^	*U*^13^	*U*^23^
S1A	0.0254 (4)	0.0331 (6)	0.0167 (5)	−0.0001 (4)	0.0001 (4)	0.0019 (5)
O1A	0.0268 (15)	0.061 (2)	0.0140 (15)	0.0078 (15)	0.0072 (13)	0.0040 (16)
O2A	0.0265 (14)	0.049 (2)	0.0195 (15)	0.0074 (14)	−0.0059 (13)	−0.0006 (15)
N1A	0.0252 (17)	0.036 (2)	0.0126 (17)	0.0037 (15)	−0.0012 (15)	0.0035 (16)
N2A	0.0258 (17)	0.050 (3)	0.0170 (19)	0.0041 (18)	0.0003 (14)	0.0097 (18)
N3A	0.0286 (18)	0.051 (3)	0.025 (2)	0.0004 (18)	0.0037 (17)	0.016 (2)
N4A	0.0268 (18)	0.0291 (19)	0.0302 (19)	0.0012 (15)	0.0000 (17)	0.0062 (16)
C1A	0.0254 (19)	0.023 (2)	0.019 (2)	−0.0022 (16)	0.0013 (16)	0.0030 (18)
C2A	0.033 (2)	0.022 (2)	0.015 (2)	−0.0017 (18)	−0.0008 (17)	−0.0009 (17)
C3A	0.027 (2)	0.020 (2)	0.024 (2)	−0.0053 (17)	−0.0029 (17)	−0.0007 (18)
C4A	0.036 (2)	0.022 (2)	0.015 (2)	−0.0051 (18)	0.0026 (18)	0.0012 (18)
C5A	0.024 (2)	0.029 (2)	0.030 (2)	−0.0031 (17)	0.0077 (18)	−0.004 (2)
C6A	0.026 (2)	0.030 (2)	0.026 (2)	0.0005 (19)	−0.0021 (16)	−0.004 (2)
C7A	0.029 (2)	0.028 (2)	0.024 (2)	0.0006 (18)	−0.0023 (17)	−0.0014 (19)
C8A	0.029 (2)	0.021 (2)	0.018 (2)	−0.0023 (17)	0.0012 (16)	−0.0041 (18)
C9A	0.029 (2)	0.020 (2)	0.0124 (19)	−0.0040 (18)	−0.0033 (15)	0.0030 (17)
C10A	0.0253 (19)	0.018 (2)	0.020 (2)	0.0006 (16)	−0.0028 (17)	−0.0017 (18)
C11A	0.0185 (18)	0.033 (2)	0.016 (2)	0.0016 (18)	0.0017 (15)	0.0023 (19)
C12A	0.0260 (19)	0.021 (2)	0.020 (2)	−0.0018 (17)	−0.0037 (16)	0.0065 (19)
C13A	0.0245 (19)	0.023 (2)	0.022 (2)	0.0034 (16)	0.0028 (17)	0.0009 (19)
C14A	0.034 (2)	0.026 (2)	0.0104 (19)	0.0022 (18)	−0.0048 (16)	0.0036 (18)
C15A	0.023 (2)	0.028 (2)	0.030 (2)	0.0047 (19)	0.0015 (18)	0.005 (2)
C16A	0.031 (2)	0.033 (2)	0.017 (2)	0.0018 (19)	0.0050 (18)	0.0025 (19)
C17A	0.027 (2)	0.027 (2)	0.025 (2)	0.0001 (18)	0.0028 (17)	0.0067 (19)
C18A	0.028 (2)	0.053 (3)	0.032 (3)	0.007 (2)	0.008 (2)	0.016 (2)
C19A	0.037 (2)	0.050 (3)	0.013 (2)	0.008 (2)	−0.0022 (18)	−0.004 (2)
S1B	0.0225 (4)	0.0342 (6)	0.0167 (5)	−0.0002 (4)	−0.0002 (4)	0.0009 (5)
O1B	0.0211 (14)	0.065 (2)	0.0322 (19)	0.0067 (16)	0.0042 (14)	0.0094 (18)
O2B	0.0268 (14)	0.0429 (19)	0.0220 (15)	0.0032 (14)	−0.0041 (13)	−0.0040 (15)
N1B	0.0236 (17)	0.031 (2)	0.0200 (19)	0.0006 (14)	0.0013 (16)	−0.0029 (17)
N2B	0.0260 (18)	0.052 (3)	0.0183 (19)	0.0023 (18)	0.0002 (14)	0.0063 (19)
N3B	0.0288 (18)	0.045 (2)	0.025 (2)	0.0006 (17)	−0.0004 (17)	0.012 (2)
N4B	0.0288 (18)	0.0294 (19)	0.0198 (17)	−0.0002 (15)	−0.0006 (15)	0.0044 (15)
C1B	0.026 (2)	0.023 (2)	0.016 (2)	0.0008 (17)	−0.0020 (15)	−0.0066 (17)
C2B	0.0201 (19)	0.025 (2)	0.028 (2)	−0.0035 (17)	0.0008 (17)	0.0008 (19)
C3B	0.027 (2)	0.022 (2)	0.016 (2)	−0.0023 (17)	0.0010 (16)	−0.0013 (18)
C4B	0.026 (2)	0.022 (2)	0.028 (2)	−0.0029 (17)	0.0007 (18)	0.0005 (19)
C5B	0.025 (2)	0.028 (2)	0.021 (2)	−0.0074 (17)	0.0018 (17)	−0.0008 (19)
C6B	0.0210 (18)	0.029 (2)	0.031 (2)	−0.0004 (18)	−0.0029 (16)	−0.002 (2)
C7B	0.029 (2)	0.030 (2)	0.019 (2)	−0.0010 (19)	−0.0023 (16)	−0.0011 (19)
C8B	0.0221 (19)	0.029 (2)	0.024 (2)	−0.0014 (17)	−0.0006 (16)	0.0004 (19)
C9B	0.025 (2)	0.024 (2)	0.020 (2)	0.0000 (18)	−0.0028 (17)	−0.0047 (19)
C10B	0.025 (2)	0.022 (2)	0.017 (2)	0.0004 (16)	0.0008 (16)	0.0007 (17)
C11B	0.0200 (18)	0.024 (2)	0.022 (2)	−0.0014 (18)	0.0021 (15)	0.0006 (18)
C12B	0.0215 (18)	0.021 (2)	0.017 (2)	0.0015 (16)	0.0018 (16)	−0.0012 (18)
C13B	0.0259 (19)	0.024 (2)	0.017 (2)	0.0009 (16)	−0.0008 (17)	0.0019 (18)
C14B	0.024 (2)	0.0194 (19)	0.035 (3)	−0.0005 (17)	−0.0026 (18)	−0.001 (2)
C15B	0.0230 (19)	0.030 (2)	0.021 (2)	0.0012 (18)	−0.0025 (17)	0.0029 (19)
C16B	0.0230 (19)	0.028 (2)	0.028 (2)	0.0013 (17)	0.0040 (17)	0.0082 (19)
C17B	0.026 (2)	0.031 (2)	0.0124 (18)	0.0000 (18)	0.0008 (15)	0.0010 (17)
C18B	0.025 (2)	0.061 (3)	0.029 (2)	0.009 (2)	0.0073 (19)	0.011 (2)
C19B	0.029 (2)	0.049 (3)	0.029 (3)	0.006 (2)	−0.0046 (19)	−0.007 (2)
C1SA	0.070 (4)	0.045 (4)	0.091 (6)	0.008 (3)	−0.055 (4)	−0.004 (4)
O1SA	0.0391 (17)	0.0270 (18)	0.054 (2)	0.0036 (15)	−0.0169 (17)	−0.0028 (17)
C1SB	0.062 (4)	0.033 (3)	0.101 (6)	0.001 (3)	−0.051 (4)	−0.002 (4)
O1SB	0.0427 (18)	0.0318 (19)	0.043 (2)	0.0008 (16)	−0.0132 (16)	−0.0011 (17)

### (I) (Z)-5-[2-(Benzo[b]thiophen-2-yl)-1-(3,5-dimethoxyphenyl)ethenyl]-1H-tetrazole methanol monosolvate Geometric parameters (Å, º)

**Table:**

S1A—C8A	1.742 (4)	N1B—C11B	1.339 (6)
S1A—C1A	1.768 (4)	N1B—N2B	1.346 (5)
O1A—C16A	1.385 (5)	N1B—H1NB	0.8800
O1A—C18A	1.422 (5)	N2B—N3B	1.281 (6)
O2A—C14A	1.379 (5)	N3B—N4B	1.360 (6)
O2A—C19A	1.437 (5)	N4B—C11B	1.319 (6)
N1A—C11A	1.317 (6)	N4B—H4NB	0.8800
N1A—N2A	1.365 (5)	C1B—C2B	1.376 (6)
N1A—H1NA	0.8800	C1B—C9B	1.444 (6)
N2A—N3A	1.313 (7)	C2B—C3B	1.433 (6)
N3A—N4A	1.358 (6)	C2B—H2B	0.9500
N4A—C11A	1.337 (7)	C3B—C8B	1.406 (6)
N4A—H4NA	0.8800	C3B—C4B	1.408 (6)
C1A—C2A	1.348 (6)	C4B—C5B	1.391 (6)
C1A—C9A	1.449 (6)	C4B—H4B	0.9500
C2A—C3A	1.418 (6)	C5B—C6B	1.386 (6)
C2A—H2A	0.9500	C5B—H5B	0.9500
C3A—C4A	1.400 (7)	C6B—C7B	1.383 (6)
C3A—C8A	1.413 (6)	C6B—H6B	0.9500
C4A—C5A	1.364 (6)	C7B—C8B	1.395 (6)
C4A—H4A	0.9500	C7B—H7B	0.9500
C5A—C6A	1.419 (7)	C9B—C10B	1.351 (6)
C5A—H5A	0.9500	C9B—H9B	0.9500
C6A—C7A	1.372 (6)	C10B—C11B	1.469 (6)
C6A—H6A	0.9500	C10B—C12B	1.494 (5)
C7A—C8A	1.389 (6)	C12B—C13B	1.372 (6)
C7A—H7A	0.9500	C12B—C17B	1.416 (6)
C9A—C10A	1.338 (6)	C13B—C14B	1.386 (6)
C9A—H9A	0.9500	C13B—H13B	0.9500
C10A—C12A	1.469 (6)	C14B—C15B	1.400 (7)
C10A—C11A	1.491 (6)	C15B—C16B	1.400 (6)
C12A—C17A	1.363 (6)	C15B—H15B	0.9500
C12A—C13A	1.431 (6)	C16B—C17B	1.398 (6)
C13A—C14A	1.378 (6)	C17B—H17B	0.9500
C13A—H13A	0.9500	C18B—H18D	0.9800
C14A—C15A	1.396 (6)	C18B—H18E	0.9800
C15A—C16A	1.359 (6)	C18B—H18F	0.9800
C15A—H15A	0.9500	C19B—H19D	0.9800
C16A—C17A	1.394 (6)	C19B—H19E	0.9800
C17A—H17A	0.9500	C19B—H19F	0.9800
C18A—H18A	0.9800	C1SA—O1SA	1.421 (7)
C18A—H18B	0.9800	C1SA—H1S1	0.9800
C18A—H18C	0.9800	C1SA—H1S2	0.9800
C19A—H19A	0.9800	C1SA—H1S3	0.9800
C19A—H19B	0.9800	O1SA—H1SA	0.8400
C19A—H19C	0.9800	O1SA—H2SA	0.8400
S1B—C8B	1.733 (4)	C1SB—O1SB	1.409 (6)
S1B—C1B	1.750 (4)	C1SB—H1S4	0.9800
O1B—C16B	1.345 (5)	C1SB—H1S5	0.9800
O1B—C18B	1.425 (5)	C1SB—H1S6	0.9800
O2B—C14B	1.355 (6)	O1SB—H1SB	0.8400
O2B—C19B	1.415 (6)	O1SB—H2SB	0.8400
			
C8A—S1A—C1A	91.3 (2)	C11B—N4B—N3B	108.9 (4)
C16A—O1A—C18A	116.9 (4)	C11B—N4B—H4NB	125.5
C14A—O2A—C19A	117.7 (3)	N3B—N4B—H4NB	125.5
C11A—N1A—N2A	108.2 (4)	C2B—C1B—C9B	122.9 (4)
C11A—N1A—H1NA	125.9	C2B—C1B—S1B	111.2 (3)
N2A—N1A—H1NA	125.9	C9B—C1B—S1B	125.9 (3)
N3A—N2A—N1A	106.8 (4)	C1B—C2B—C3B	113.7 (4)
N2A—N3A—N4A	109.6 (4)	C1B—C2B—H2B	123.1
C11A—N4A—N3A	106.2 (4)	C3B—C2B—H2B	123.1
C11A—N4A—H4NA	126.9	C8B—C3B—C4B	119.8 (4)
N3A—N4A—H4NA	126.9	C8B—C3B—C2B	111.5 (4)
C2A—C1A—C9A	124.6 (4)	C4B—C3B—C2B	128.6 (4)
C2A—C1A—S1A	110.8 (3)	C5B—C4B—C3B	117.6 (4)
C9A—C1A—S1A	124.6 (3)	C5B—C4B—H4B	121.2
C1A—C2A—C3A	115.3 (4)	C3B—C4B—H4B	121.2
C1A—C2A—H2A	122.4	C6B—C5B—C4B	121.9 (4)
C3A—C2A—H2A	122.4	C6B—C5B—H5B	119.1
C4A—C3A—C8A	118.2 (4)	C4B—C5B—H5B	119.1
C4A—C3A—C2A	130.4 (4)	C7B—C6B—C5B	121.3 (4)
C8A—C3A—C2A	111.4 (4)	C7B—C6B—H6B	119.4
C5A—C4A—C3A	120.6 (4)	C5B—C6B—H6B	119.4
C5A—C4A—H4A	119.7	C6B—C7B—C8B	117.8 (4)
C3A—C4A—H4A	119.7	C6B—C7B—H7B	121.1
C4A—C5A—C6A	120.1 (4)	C8B—C7B—H7B	121.1
C4A—C5A—H5A	120.0	C7B—C8B—C3B	121.6 (4)
C6A—C5A—H5A	120.0	C7B—C8B—S1B	126.7 (4)
C7A—C6A—C5A	120.8 (4)	C3B—C8B—S1B	111.7 (3)
C7A—C6A—H6A	119.6	C10B—C9B—C1B	129.6 (4)
C5A—C6A—H6A	119.6	C10B—C9B—H9B	115.2
C6A—C7A—C8A	118.6 (4)	C1B—C9B—H9B	115.2
C6A—C7A—H7A	120.7	C9B—C10B—C11B	120.1 (4)
C8A—C7A—H7A	120.7	C9B—C10B—C12B	123.0 (4)
C7A—C8A—C3A	121.7 (4)	C11B—C10B—C12B	116.9 (4)
C7A—C8A—S1A	127.1 (3)	N4B—C11B—N1B	107.2 (4)
C3A—C8A—S1A	111.2 (3)	N4B—C11B—C10B	127.0 (4)
C10A—C9A—C1A	131.2 (4)	N1B—C11B—C10B	125.8 (4)
C10A—C9A—H9A	114.4	C13B—C12B—C17B	120.9 (4)
C1A—C9A—H9A	114.4	C13B—C12B—C10B	121.3 (4)
C9A—C10A—C12A	124.7 (4)	C17B—C12B—C10B	117.7 (4)
C9A—C10A—C11A	120.1 (4)	C12B—C13B—C14B	119.9 (4)
C12A—C10A—C11A	115.1 (4)	C12B—C13B—H13B	120.0
N1A—C11A—N4A	109.2 (4)	C14B—C13B—H13B	120.0
N1A—C11A—C10A	127.6 (4)	O2B—C14B—C13B	125.1 (4)
N4A—C11A—C10A	123.2 (4)	O2B—C14B—C15B	114.3 (4)
C17A—C12A—C13A	118.3 (4)	C13B—C14B—C15B	120.6 (5)
C17A—C12A—C10A	121.9 (4)	C14B—C15B—C16B	119.6 (4)
C13A—C12A—C10A	119.8 (4)	C14B—C15B—H15B	120.2
C14A—C13A—C12A	118.5 (4)	C16B—C15B—H15B	120.2
C14A—C13A—H13A	120.8	O1B—C16B—C17B	115.2 (4)
C12A—C13A—H13A	120.8	O1B—C16B—C15B	124.7 (4)
C13A—C14A—O2A	123.3 (4)	C17B—C16B—C15B	120.1 (4)
C13A—C14A—C15A	122.5 (4)	C16B—C17B—C12B	118.8 (4)
O2A—C14A—C15A	114.2 (4)	C16B—C17B—H17B	120.6
C16A—C15A—C14A	118.0 (4)	C12B—C17B—H17B	120.6
C16A—C15A—H15A	121.0	O1B—C18B—H18D	109.5
C14A—C15A—H15A	121.0	O1B—C18B—H18E	109.5
C15A—C16A—O1A	124.0 (4)	H18D—C18B—H18E	109.5
C15A—C16A—C17A	121.1 (4)	O1B—C18B—H18F	109.5
O1A—C16A—C17A	114.9 (4)	H18D—C18B—H18F	109.5
C12A—C17A—C16A	121.7 (4)	H18E—C18B—H18F	109.5
C12A—C17A—H17A	119.2	O2B—C19B—H19D	109.5
C16A—C17A—H17A	119.2	O2B—C19B—H19E	109.5
O1A—C18A—H18A	109.5	H19D—C19B—H19E	109.5
O1A—C18A—H18B	109.5	O2B—C19B—H19F	109.5
H18A—C18A—H18B	109.5	H19D—C19B—H19F	109.5
O1A—C18A—H18C	109.5	H19E—C19B—H19F	109.5
H18A—C18A—H18C	109.5	O1SA—C1SA—H1S1	109.5
H18B—C18A—H18C	109.5	O1SA—C1SA—H1S2	109.5
O2A—C19A—H19A	109.5	H1S1—C1SA—H1S2	109.5
O2A—C19A—H19B	109.5	O1SA—C1SA—H1S3	109.5
H19A—C19A—H19B	109.5	H1S1—C1SA—H1S3	109.5
O2A—C19A—H19C	109.5	H1S2—C1SA—H1S3	109.5
H19A—C19A—H19C	109.5	C1SA—O1SA—H1SA	109.5
H19B—C19A—H19C	109.5	C1SA—O1SA—H2SA	109.5
C8B—S1B—C1B	91.9 (2)	O1SB—C1SB—H1S4	109.5
C16B—O1B—C18B	118.0 (4)	O1SB—C1SB—H1S5	109.5
C14B—O2B—C19B	117.2 (3)	H1S4—C1SB—H1S5	109.5
C11B—N1B—N2B	106.8 (4)	O1SB—C1SB—H1S6	109.5
C11B—N1B—H1NB	126.6	H1S4—C1SB—H1S6	109.5
N2B—N1B—H1NB	126.6	H1S5—C1SB—H1S6	109.5
N3B—N2B—N1B	110.4 (4)	C1SB—O1SB—H1SB	109.5
N2B—N3B—N4B	106.7 (4)	C1SB—O1SB—H2SB	109.5
			
C11A—N1A—N2A—N3A	−0.6 (4)	C11B—N1B—N2B—N3B	−0.8 (5)
N1A—N2A—N3A—N4A	0.9 (5)	N1B—N2B—N3B—N4B	0.4 (5)
N2A—N3A—N4A—C11A	−0.8 (5)	N2B—N3B—N4B—C11B	0.2 (5)
C8A—S1A—C1A—C2A	−1.1 (4)	C8B—S1B—C1B—C2B	0.5 (4)
C8A—S1A—C1A—C9A	178.9 (4)	C8B—S1B—C1B—C9B	179.6 (4)
C9A—C1A—C2A—C3A	−178.9 (4)	C9B—C1B—C2B—C3B	−179.6 (4)
S1A—C1A—C2A—C3A	1.1 (5)	S1B—C1B—C2B—C3B	−0.4 (5)
C1A—C2A—C3A—C4A	−179.1 (5)	C1B—C2B—C3B—C8B	0.1 (6)
C1A—C2A—C3A—C8A	−0.4 (6)	C1B—C2B—C3B—C4B	179.9 (4)
C8A—C3A—C4A—C5A	1.1 (7)	C8B—C3B—C4B—C5B	−0.1 (7)
C2A—C3A—C4A—C5A	179.7 (5)	C2B—C3B—C4B—C5B	−180.0 (5)
C3A—C4A—C5A—C6A	−0.7 (7)	C3B—C4B—C5B—C6B	−0.3 (7)
C4A—C5A—C6A—C7A	−1.2 (7)	C4B—C5B—C6B—C7B	0.4 (8)
C5A—C6A—C7A—C8A	2.6 (7)	C5B—C6B—C7B—C8B	−0.1 (7)
C6A—C7A—C8A—C3A	−2.2 (7)	C6B—C7B—C8B—C3B	−0.3 (7)
C6A—C7A—C8A—S1A	−180.0 (4)	C6B—C7B—C8B—S1B	179.7 (4)
C4A—C3A—C8A—C7A	0.4 (7)	C4B—C3B—C8B—C7B	0.4 (7)
C2A—C3A—C8A—C7A	−178.5 (4)	C2B—C3B—C8B—C7B	−179.7 (4)
C4A—C3A—C8A—S1A	178.4 (3)	C4B—C3B—C8B—S1B	−179.6 (4)
C2A—C3A—C8A—S1A	−0.4 (5)	C2B—C3B—C8B—S1B	0.3 (5)
C1A—S1A—C8A—C7A	178.8 (5)	C1B—S1B—C8B—C7B	179.6 (5)
C1A—S1A—C8A—C3A	0.8 (3)	C1B—S1B—C8B—C3B	−0.4 (4)
C2A—C1A—C9A—C10A	176.3 (5)	C2B—C1B—C9B—C10B	−176.6 (5)
S1A—C1A—C9A—C10A	−3.6 (8)	S1B—C1B—C9B—C10B	4.3 (8)
C1A—C9A—C10A—C12A	179.7 (5)	C1B—C9B—C10B—C11B	−0.1 (8)
C1A—C9A—C10A—C11A	−2.2 (8)	C1B—C9B—C10B—C12B	−178.6 (5)
N2A—N1A—C11A—N4A	0.0 (4)	N3B—N4B—C11B—N1B	−0.7 (5)
N2A—N1A—C11A—C10A	178.6 (4)	N3B—N4B—C11B—C10B	179.4 (4)
N3A—N4A—C11A—N1A	0.5 (4)	N2B—N1B—C11B—N4B	0.9 (4)
N3A—N4A—C11A—C10A	−178.2 (3)	N2B—N1B—C11B—C10B	−179.2 (4)
C9A—C10A—C11A—N1A	−90.9 (5)	C9B—C10B—C11B—N4B	−90.0 (6)
C12A—C10A—C11A—N1A	87.3 (5)	C12B—C10B—C11B—N4B	88.6 (5)
C9A—C10A—C11A—N4A	87.5 (5)	C9B—C10B—C11B—N1B	90.1 (5)
C12A—C10A—C11A—N4A	−94.3 (5)	C12B—C10B—C11B—N1B	−91.3 (5)
C9A—C10A—C12A—C17A	159.7 (5)	C9B—C10B—C12B—C13B	19.7 (7)
C11A—C10A—C12A—C17A	−18.4 (6)	C11B—C10B—C12B—C13B	−158.9 (4)
C9A—C10A—C12A—C13A	−20.2 (7)	C9B—C10B—C12B—C17B	−159.0 (5)
C11A—C10A—C12A—C13A	161.6 (4)	C11B—C10B—C12B—C17B	22.5 (6)
C17A—C12A—C13A—C14A	−2.2 (6)	C17B—C12B—C13B—C14B	0.7 (7)
C10A—C12A—C13A—C14A	177.7 (4)	C10B—C12B—C13B—C14B	−177.9 (4)
C12A—C13A—C14A—O2A	−178.9 (4)	C19B—O2B—C14B—C13B	−7.5 (7)
C12A—C13A—C14A—C15A	2.0 (7)	C19B—O2B—C14B—C15B	171.4 (5)
C19A—O2A—C14A—C13A	6.4 (7)	C12B—C13B—C14B—O2B	179.0 (4)
C19A—O2A—C14A—C15A	−174.5 (4)	C12B—C13B—C14B—C15B	0.3 (7)
C13A—C14A—C15A—C16A	−1.2 (7)	O2B—C14B—C15B—C16B	−179.3 (4)
O2A—C14A—C15A—C16A	179.7 (4)	C13B—C14B—C15B—C16B	−0.4 (7)
C14A—C15A—C16A—O1A	−178.8 (4)	C18B—O1B—C16B—C17B	179.5 (5)
C14A—C15A—C16A—C17A	0.5 (7)	C18B—O1B—C16B—C15B	−0.2 (7)
C18A—O1A—C16A—C15A	0.5 (7)	C14B—C15B—C16B—O1B	179.3 (4)
C18A—O1A—C16A—C17A	−178.9 (4)	C14B—C15B—C16B—C17B	−0.4 (7)
C13A—C12A—C17A—C16A	1.7 (7)	O1B—C16B—C17B—C12B	−178.4 (4)
C10A—C12A—C17A—C16A	−178.3 (4)	C15B—C16B—C17B—C12B	1.3 (7)
C15A—C16A—C17A—C12A	−0.8 (8)	C13B—C12B—C17B—C16B	−1.4 (7)
O1A—C16A—C17A—C12A	178.6 (4)	C10B—C12B—C17B—C16B	177.2 (4)

### (I) (Z)-5-[2-(Benzo[b]thiophen-2-yl)-1-(3,5-dimethoxyphenyl)ethenyl]-1H-tetrazole methanol monosolvate Hydrogen-bond geometry (Å, º)

**Table:**

*D*—H···*A*	*D*—H	H···*A*	*D*···*A*	*D*—H···*A*
N1*A*—H1*NA*···N1*A*^i^	0.88	1.91	2.787 (9)	176
N4*A*—H4*NA*···O1*SB*	0.88	1.87	2.736 (6)	168
N1*B*—H1*NB*···N1*B*^ii^	0.88	1.92	2.792 (9)	174
N4*B*—H4*NB*···O1*SA*	0.88	1.91	2.769 (6)	165
O1*SA*—H1*SA*···N4*B*	0.84	1.97	2.769 (6)	158
O1*SA*—H2*SA*···O1*SA*^iii^	0.84	1.81	2.646 (7)	177
O1*SB*—H1*SB*···N4*A*	0.84	1.90	2.736 (6)	176

Symmetry codes: (i) −*x*, −*y*, *z*; (ii) −*x*+1, −*y*+1, *z*; (iii) −*x*+1, −*y*, *z*.

### (II) (Z)-5-[2-(Benzo[b]thiophen-3-yl)-1-(3,4,5-trimethoxyphenyl)ethenyl]-1H-tetrazole Crystal data

**Table:**

C_20_H_18_N_4_O_3_S	*F*(000) = 824
*M**_r_* = 394.44	*D*_x_ = 1.393 Mg m^−^^3^
Monoclinic, *P*2_1_/*c*	Cu *K*α radiation, λ = 1.54178 Å
*a* = 4.8888 (1) Å	Cell parameters from 9924 reflections
*b* = 24.6650 (6) Å	θ = 3.4–68.3°
*c* = 15.5956 (4) Å	µ = 1.78 mm^−^^1^
β = 91.031 (1)°	*T* = 90 K
*V* = 1880.25 (8) Å^3^	Plate, colourless
*Z* = 4	0.10 × 0.08 × 0.02 mm

### (II) (Z)-5-[2-(Benzo[b]thiophen-3-yl)-1-(3,4,5-trimethoxyphenyl)ethenyl]-1H-tetrazole Data collection

**Table:**

Bruker X8 Proteum diffractometer	3337 independent reflections
Radiation source: fine-focus rotating anode	3138 reflections with *I* > 2σ(*I*)
Detector resolution: 5.6 pixels mm^-1^	*R*_int_ = 0.037
φ and ω scans	θ_max_ = 68.5°, θ_min_ = 3.4°
Absorption correction: multi-scan (*SADABS*; Krause *et al.*, 2015)	*h* = −5→2
*T*_min_ = 0.693, *T*_max_ = 0.897	*k* = −29→29
23250 measured reflections	*l* = −18→18

### (II) (Z)-5-[2-(Benzo[b]thiophen-3-yl)-1-(3,4,5-trimethoxyphenyl)ethenyl]-1H-tetrazole Refinement

**Table:**

Refinement on *F*^2^	Primary atom site location: structure-invariant direct methods
Least-squares matrix: full	Secondary atom site location: difference Fourier map
*R*[*F*^2^ > 2σ(*F*^2^)] = 0.034	Hydrogen site location: mixed
*wR*(*F*^2^) = 0.094	H atoms treated by a mixture of independent and constrained refinement
*S* = 1.13	*w* = 1/[σ^2^(*F*_o_^2^) + (0.0391*P*)^2^ + 1.3628*P*] where *P* = (*F*_o_^2^ + 2*F*_c_^2^)/3
3337 reflections	(Δ/σ)_max_ = 0.005
259 parameters	Δρ_max_ = 0.27 e Å^−^^3^
0 restraints	Δρ_min_ = −0.31 e Å^−^^3^

### (II) (Z)-5-[2-(Benzo[b]thiophen-3-yl)-1-(3,4,5-trimethoxyphenyl)ethenyl]-1H-tetrazole Special details

**Table:**

Experimental. The crystal was mounted using polyisobutene oil on the tip of a fine glass fibre, which was fastened in a copper mounting pin with electrical solder. It was placed directly into the cold gas stream of a liquid-nitrogen based cryostat. Diffraction data were collected with the crystal at 90K, which is standard practice in this laboratory for the majority of flash-cooled crystals.
Geometry. All esds (except the esd in the dihedral angle between two l.s. planes) are estimated using the full covariance matrix. The cell esds are taken into account individually in the estimation of esds in distances, angles and torsion angles; correlations between esds in cell parameters are only used when they are defined by crystal symmetry. An approximate (isotropic) treatment of cell esds is used for estimating esds involving l.s. planes.
Refinement. Refinement progress was checked using *Platon* (Spek, 2009) and by an *R*-tensor (Parkin, 2000). The final model was further checked with the IUCr utility *checkCIF*.

### (II) (Z)-5-[2-(Benzo[b]thiophen-3-yl)-1-(3,4,5-trimethoxyphenyl)ethenyl]-1H-tetrazole Fractional atomic coordinates and isotropic or equivalent isotropic displacement parameters (Å^2^)

**Table:**

	*x*	*y*	*z*	*U*_iso_*/*U*_eq_	
S1	0.58440 (8)	0.92397 (2)	0.22030 (3)	0.02003 (13)	
N1	0.5809 (3)	0.76447 (5)	0.36657 (9)	0.0158 (3)	
H1N	0.397 (4)	0.7636 (8)	0.3563 (12)	0.019*	
N2	0.6748 (3)	0.78140 (6)	0.44376 (9)	0.0189 (3)	
N3	0.9385 (3)	0.78051 (6)	0.44059 (9)	0.0188 (3)	
N4	1.0187 (3)	0.76305 (5)	0.36194 (9)	0.0167 (3)	
C1	0.7332 (3)	0.86078 (7)	0.22620 (11)	0.0195 (3)	
H1	0.8771	0.8522	0.2657	0.023*	
C2	0.6269 (3)	0.82413 (7)	0.1694 (1)	0.0169 (3)	
C3	0.4172 (3)	0.84839 (7)	0.11505 (10)	0.0162 (3)	
C4	0.2612 (3)	0.82496 (7)	0.04825 (10)	0.0185 (3)	
H4	0.2885	0.7881	0.0327	0.022*	
C5	0.0676 (3)	0.85608 (7)	0.00545 (11)	0.0225 (4)	
H5	−0.0373	0.8405	−0.0401	0.027*	
C6	0.0238 (3)	0.91033 (7)	0.02841 (12)	0.0230 (4)	
H6	−0.1108	0.9310	−0.0018	0.028*	
C7	0.1728 (3)	0.93419 (7)	0.09405 (11)	0.0207 (4)	
H7	0.1422	0.9709	0.1098	0.025*	
C8	0.3703 (3)	0.90284 (7)	0.13672 (11)	0.0178 (3)	
C9	0.7039 (3)	0.76681 (6)	0.1624 (1)	0.0165 (3)	
H9	0.7003	0.7515	0.1065	0.020*	
C10	0.7788 (3)	0.73397 (6)	0.22705 (10)	0.0148 (3)	
C11	0.7926 (3)	0.75371 (6)	0.31634 (10)	0.0134 (3)	
C12	0.8405 (3)	0.67535 (6)	0.21635 (10)	0.0154 (3)	
C13	1.0177 (3)	0.65742 (6)	0.15372 (10)	0.0161 (3)	
H13	1.1099	0.6827	0.1186	0.019*	
C14	1.0588 (3)	0.60179 (7)	0.14293 (10)	0.0167 (3)	
C15	0.9205 (3)	0.56460 (7)	0.19391 (11)	0.0175 (3)	
C16	0.7445 (3)	0.58324 (7)	0.25707 (11)	0.0170 (3)	
C17	0.7064 (3)	0.63845 (7)	0.26888 (10)	0.0166 (3)	
H17	0.5894	0.6511	0.3126	0.020*	
O1	1.2285 (2)	0.57947 (5)	0.08429 (8)	0.0210 (3)	
C18	1.3890 (3)	0.61614 (7)	0.03497 (11)	0.0211 (4)	
H18A	1.2677	0.6393	0.0002	0.032*	
H18B	1.5088	0.5955	−0.0027	0.032*	
H18C	1.5006	0.6387	0.0736	0.032*	
O2	0.9650 (2)	0.50992 (5)	0.18551 (8)	0.0236 (3)	
C19	0.8111 (4)	0.48639 (8)	0.11584 (12)	0.0308 (4)	
H19A	0.6154	0.4924	0.1246	0.046*	
H19B	0.8476	0.4474	0.1134	0.046*	
H19C	0.8650	0.5033	0.0619	0.046*	
O3	0.6211 (2)	0.54394 (5)	0.30402 (8)	0.0226 (3)	
C20	0.4227 (3)	0.56095 (7)	0.36440 (11)	0.0220 (4)	
H20A	0.5106	0.5841	0.4079	0.033*	
H20B	0.3436	0.5291	0.3921	0.033*	
H20C	0.2776	0.5814	0.3346	0.033*	

### (II) (Z)-5-[2-(Benzo[b]thiophen-3-yl)-1-(3,4,5-trimethoxyphenyl)ethenyl]-1H-tetrazole Atomic displacement parameters (Å^2^)

**Table:**

	*U*^11^	*U*^22^	*U*^33^	*U*^12^	*U*^13^	*U*^23^
S1	0.0226 (2)	0.0163 (2)	0.0211 (2)	0.00207 (14)	−0.00164 (16)	−0.00240 (15)
N1	0.0109 (7)	0.0222 (7)	0.0142 (7)	0.0006 (5)	0.0001 (5)	−0.0018 (5)
N2	0.0157 (7)	0.0246 (7)	0.0163 (7)	0.0013 (5)	0.0004 (5)	−0.0026 (6)
N3	0.0158 (7)	0.0242 (7)	0.0163 (7)	0.0007 (5)	−0.0002 (5)	−0.0029 (6)
N4	0.0143 (7)	0.0214 (7)	0.0143 (7)	0.0004 (5)	−0.0001 (5)	−0.0016 (5)
C1	0.0197 (8)	0.0197 (8)	0.0189 (9)	0.0015 (6)	−0.0017 (6)	0.0008 (6)
C2	0.0166 (8)	0.0197 (8)	0.0146 (8)	0.0012 (6)	0.0033 (6)	0.0019 (6)
C3	0.0144 (7)	0.0191 (8)	0.0153 (8)	0.0000 (6)	0.0042 (6)	0.0028 (6)
C4	0.0181 (8)	0.0199 (8)	0.0176 (8)	−0.0011 (6)	0.0023 (6)	0.0000 (6)
C5	0.0184 (8)	0.0282 (9)	0.0207 (9)	−0.0025 (7)	−0.0022 (6)	0.0010 (7)
C6	0.0154 (8)	0.0263 (9)	0.0271 (10)	0.0026 (7)	−0.0013 (7)	0.0054 (7)
C7	0.0174 (8)	0.0177 (8)	0.0272 (9)	0.0020 (6)	0.0035 (6)	0.0025 (7)
C8	0.0163 (8)	0.0193 (8)	0.0180 (8)	−0.0004 (6)	0.0038 (6)	0.0007 (6)
C9	0.0163 (8)	0.0189 (8)	0.0142 (8)	−0.0003 (6)	0.0014 (6)	−0.0019 (6)
C10	0.0104 (7)	0.0184 (8)	0.0157 (8)	−0.0010 (6)	0.0015 (6)	−0.0016 (6)
C11	0.0124 (7)	0.0135 (7)	0.0144 (8)	0.0004 (5)	0.0005 (6)	0.0007 (6)
C12	0.0125 (7)	0.0178 (8)	0.0158 (8)	0.0000 (6)	−0.0034 (6)	−0.0011 (6)
C13	0.0143 (7)	0.0179 (8)	0.0162 (8)	−0.0005 (6)	−0.0012 (6)	0.0014 (6)
C14	0.0138 (7)	0.0207 (8)	0.0155 (8)	0.0020 (6)	−0.0012 (6)	−0.0026 (6)
C15	0.0174 (8)	0.0165 (8)	0.0186 (8)	0.0020 (6)	−0.0026 (6)	−0.0011 (6)
C16	0.0158 (8)	0.0185 (8)	0.0168 (8)	−0.0023 (6)	−0.0015 (6)	0.0021 (6)
C17	0.0150 (7)	0.0205 (8)	0.0142 (8)	0.0010 (6)	−0.0001 (6)	−0.0015 (6)
O1	0.0206 (6)	0.0196 (6)	0.0231 (7)	0.0018 (4)	0.0073 (5)	−0.0023 (5)
C18	0.0174 (8)	0.0254 (9)	0.0206 (9)	−0.0004 (6)	0.0047 (6)	−0.0008 (7)
O2	0.0285 (6)	0.0152 (6)	0.0270 (7)	0.0027 (5)	0.0005 (5)	−0.0012 (5)
C19	0.0457 (11)	0.0204 (9)	0.0266 (10)	−0.0074 (8)	0.0069 (8)	−0.0056 (7)
O3	0.0267 (6)	0.0173 (6)	0.0242 (7)	−0.0021 (5)	0.0084 (5)	0.0021 (5)
C20	0.0212 (8)	0.0234 (9)	0.0216 (9)	−0.0028 (7)	0.0049 (7)	0.0010 (7)

### (II) (Z)-5-[2-(Benzo[b]thiophen-3-yl)-1-(3,4,5-trimethoxyphenyl)ethenyl]-1H-tetrazole Geometric parameters (Å, º)

**Table:**

S1—C1	1.7218 (17)	C10—C12	1.487 (2)
S1—C8	1.7371 (17)	C12—C13	1.389 (2)
N1—C11	1.336 (2)	C12—C17	1.396 (2)
N1—N2	1.3470 (19)	C13—C14	1.397 (2)
N1—H1N	0.91 (2)	C13—H13	0.9500
N2—N3	1.2911 (19)	C14—O1	1.3622 (19)
N3—N4	1.3641 (19)	C14—C15	1.396 (2)
N4—C11	1.324 (2)	C15—O2	1.373 (2)
C1—C2	1.362 (2)	C15—C16	1.397 (2)
C1—H1	0.9500	C16—O3	1.362 (2)
C2—C3	1.448 (2)	C16—C17	1.387 (2)
C2—C9	1.467 (2)	C17—H17	0.9500
C3—C4	1.404 (2)	O1—C18	1.431 (2)
C3—C8	1.405 (2)	C18—H18A	0.9800
C4—C5	1.381 (2)	C18—H18B	0.9800
C4—H4	0.9500	C18—H18C	0.9800
C5—C6	1.403 (3)	O2—C19	1.433 (2)
C5—H5	0.9500	C19—H19A	0.9800
C6—C7	1.378 (3)	C19—H19B	0.9800
C6—H6	0.9500	C19—H19C	0.9800
C7—C8	1.396 (2)	O3—C20	1.427 (2)
C7—H7	0.9500	C20—H20A	0.9800
C9—C10	1.339 (2)	C20—H20B	0.9800
C9—H9	0.9500	C20—H20C	0.9800
C10—C11	1.476 (2)		
			
C1—S1—C8	90.97 (8)	C13—C12—C17	120.70 (15)
C11—N1—N2	109.29 (13)	C13—C12—C10	121.24 (14)
C11—N1—H1N	131.7 (12)	C17—C12—C10	118.02 (14)
N2—N1—H1N	119.0 (12)	C12—C13—C14	119.34 (15)
N3—N2—N1	106.55 (12)	C12—C13—H13	120.3
N2—N3—N4	110.08 (12)	C14—C13—H13	120.3
C11—N4—N3	106.69 (13)	O1—C14—C15	115.07 (14)
C2—C1—S1	114.20 (13)	O1—C14—C13	124.64 (15)
C2—C1—H1	122.9	C15—C14—C13	120.29 (15)
S1—C1—H1	122.9	O2—C15—C14	120.79 (15)
C1—C2—C3	111.37 (15)	O2—C15—C16	119.41 (15)
C1—C2—C9	126.31 (15)	C14—C15—C16	119.73 (15)
C3—C2—C9	122.31 (15)	O3—C16—C17	124.42 (15)
C4—C3—C8	118.88 (15)	O3—C16—C15	115.42 (14)
C4—C3—C2	129.32 (15)	C17—C16—C15	120.16 (15)
C8—C3—C2	111.79 (15)	C16—C17—C12	119.76 (15)
C5—C4—C3	119.22 (16)	C16—C17—H17	120.1
C5—C4—H4	120.4	C12—C17—H17	120.1
C3—C4—H4	120.4	C14—O1—C18	116.88 (13)
C4—C5—C6	120.87 (16)	O1—C18—H18A	109.5
C4—C5—H5	119.6	O1—C18—H18B	109.5
C6—C5—H5	119.6	H18A—C18—H18B	109.5
C7—C6—C5	121.07 (16)	O1—C18—H18C	109.5
C7—C6—H6	119.5	H18A—C18—H18C	109.5
C5—C6—H6	119.5	H18B—C18—H18C	109.5
C6—C7—C8	118.00 (16)	C15—O2—C19	112.84 (13)
C6—C7—H7	121.0	O2—C19—H19A	109.5
C8—C7—H7	121.0	O2—C19—H19B	109.5
C7—C8—C3	121.96 (16)	H19A—C19—H19B	109.5
C7—C8—S1	126.42 (13)	O2—C19—H19C	109.5
C3—C8—S1	111.62 (12)	H19A—C19—H19C	109.5
C10—C9—C2	126.42 (15)	H19B—C19—H19C	109.5
C10—C9—H9	116.8	C16—O3—C20	117.28 (13)
C2—C9—H9	116.8	O3—C20—H20A	109.5
C9—C10—C11	121.21 (14)	O3—C20—H20B	109.5
C9—C10—C12	123.84 (15)	H20A—C20—H20B	109.5
C11—C10—C12	114.88 (13)	O3—C20—H20C	109.5
N4—C11—N1	107.39 (14)	H20A—C20—H20C	109.5
N4—C11—C10	126.03 (14)	H20B—C20—H20C	109.5
N1—C11—C10	126.58 (14)		
			
C11—N1—N2—N3	−0.48 (17)	C9—C10—C11—N4	−107.56 (19)
N1—N2—N3—N4	−0.08 (17)	C12—C10—C11—N4	75.24 (19)
N2—N3—N4—C11	0.61 (17)	C9—C10—C11—N1	73.8 (2)
C8—S1—C1—C2	−1.09 (14)	C12—C10—C11—N1	−103.42 (18)
S1—C1—C2—C3	2.01 (18)	C9—C10—C12—C13	49.6 (2)
S1—C1—C2—C9	−177.01 (13)	C11—C10—C12—C13	−133.26 (15)
C1—C2—C3—C4	179.11 (16)	C9—C10—C12—C17	−127.84 (17)
C9—C2—C3—C4	−1.8 (3)	C11—C10—C12—C17	49.29 (19)
C1—C2—C3—C8	−2.11 (19)	C17—C12—C13—C14	0.7 (2)
C9—C2—C3—C8	176.95 (14)	C10—C12—C13—C14	−176.67 (14)
C8—C3—C4—C5	0.4 (2)	C12—C13—C14—O1	−179.49 (14)
C2—C3—C4—C5	179.09 (15)	C12—C13—C14—C15	0.8 (2)
C3—C4—C5—C6	−0.6 (2)	O1—C14—C15—O2	2.1 (2)
C4—C5—C6—C7	0.2 (3)	C13—C14—C15—O2	−178.17 (14)
C5—C6—C7—C8	0.4 (3)	O1—C14—C15—C16	178.98 (14)
C6—C7—C8—C3	−0.6 (2)	C13—C14—C15—C16	−1.3 (2)
C6—C7—C8—S1	179.37 (13)	O2—C15—C16—O3	−2.4 (2)
C4—C3—C8—C7	0.2 (2)	C14—C15—C16—O3	−179.38 (14)
C2—C3—C8—C7	−178.70 (14)	O2—C15—C16—C17	177.21 (14)
C4—C3—C8—S1	−179.77 (12)	C14—C15—C16—C17	0.3 (2)
C2—C3—C8—S1	1.32 (17)	O3—C16—C17—C12	−179.17 (14)
C1—S1—C8—C7	179.83 (15)	C15—C16—C17—C12	1.2 (2)
C1—S1—C8—C3	−0.18 (12)	C13—C12—C17—C16	−1.7 (2)
C1—C2—C9—C10	34.3 (3)	C10—C12—C17—C16	175.75 (14)
C3—C2—C9—C10	−144.62 (16)	C15—C14—O1—C18	−175.64 (14)
C2—C9—C10—C11	−0.3 (2)	C13—C14—O1—C18	4.6 (2)
C2—C9—C10—C12	176.63 (14)	C14—C15—O2—C19	−81.92 (19)
N3—N4—C11—N1	−0.88 (17)	C16—C15—O2—C19	101.17 (17)
N3—N4—C11—C10	−179.75 (14)	C17—C16—O3—C20	5.4 (2)
N2—N1—C11—N4	0.86 (17)	C15—C16—O3—C20	−174.98 (14)
N2—N1—C11—C10	179.73 (14)		

### (II) (Z)-5-[2-(Benzo[b]thiophen-3-yl)-1-(3,4,5-trimethoxyphenyl)ethenyl]-1H-tetrazole Hydrogen-bond geometry (Å, º)

**Table:**

*D*—H···*A*	*D*—H	H···*A*	*D*···*A*	*D*—H···*A*
N1—H1*N*···N3^i^	0.91 (2)	2.65 (2)	3.3886 (19)	138.5 (16)
N1—H1*N*···N4^i^	0.91 (2)	1.85 (2)	2.7482 (19)	167.1 (18)

Symmetry code: (i) *x*−1, *y*, *z*.
